# Pulmonary vein isolation in patients with paroxysmal atrial fibrillation is associated with regional cardiac sympathetic denervation

**DOI:** 10.1186/2191-219X-3-81

**Published:** 2013-12-21

**Authors:** Christian Wenning, Philipp S Lange, Christoph Schülke, Alexis Vrachimis, Gerold Mönnig, Otmar Schober, Lars Eckardt, Michael Schäfers

**Affiliations:** 1 Department of Nuclear Medicine, Münster University Hospital, Münster, Germany; 2 European Institute for Molecular Imaging, University of Münster, Münster, Germany; 3 Division of Electrophysiology, Department of Cardiology and Angiology, Münster University Hospital, Münster, Germany; 4 Department of Clinical Radiology, Münster University Hospital, Münster, Germany; 5 Cluster of Excellence EXC 1003 ‘Cells in Motion’, Münster, Germany

**Keywords:** Atrial fibrillation, Pulmonary vein isolation, Sympathetic nervous system, Imaging, Single-photon emission computed tomography

## Abstract

**Background:**

Circumferential pulmonary vein isolation (PVI) is the cornerstone of the current state-of-the-art management of atrial fibrillation (AF). However, the precise mechanisms behind AF relapses post PVI are still unknown. Since the activity of the autonomous nervous system is crucial in triggering paroxysmal AF, we hypothesized that PVI is associated with changes of cardiac sympathetic activity.

**Methods:**

Sixteen patients with paroxysmal AF underwent cardiac iodine-123-meta-iodobenzylguanidine (^123^I-mIBG) planar cardiac imaging and single-photon emission computed tomography with low-dose computed tomography (SPECT/CT) for attenuation correction before and 4 weeks after PVI. The heart-to-mediastinum ratio (H/M ratio), washout rate (WR), regional myocardial uptake, and regional washout were analyzed.

**Results:**

The late H/M ratio was unchanged by PVI (pre, 2.9 ± 0.5 vs. post, 2.7 ± 0.6, *p* = 0.53). Four of the 16 patients (25%) displayed regional deficits before PVI. After PVI, regional deficits were present in ten patients (62.5%) with newly emerging deficits localized in the inferolateral wall. In a 6-month follow-up, four out of the ten patients (40%) with regional ^123^I-mIBG defects suffered from a recurrence of AF, while only one of the six patients (16.7%) without a regional ^123^I-mIBG defect experienced a recurrence.

**Conclusion:**

A significant number of patients with paroxysmal AF show regional cardiac sympathetic innervation deficits at baseline. In addition, PVI is associated with newly emerging defects. The presence of regional sympathetic denervation after PVI may correlate with the risk of AF relapses.

## Background

Atrial fibrillation (AF) is the most common arrhythmia in industrialized countries with an increasing burden of morbidity. Circumferential pulmonary vein isolation (PVI) is a cornerstone of modern AF management showing success rates of up to 60 to 70% [1,2]. However, a significant number of patients suffer from a relapse of AF after PVI [2,3]. Clinical parameters such as the type of AF, age, and the left atrial diameter correlate with the outcome after PVI in AF patients. However, the prediction for the individual patient is not yet established in clinical algorithms [1]. Advanced imaging methods such as delayed enhancement magnetic resonance imaging of the left atrium are currently being developed but are cumbersome and have not yet been tested in large multicenter clinical trials [4-7]. Alternative imaging modalities might provide novel insights into the mechanisms that orchestrate relapses in patients with AF and may be helpful for the prediction of the individual outcome following PVI. Besides fibrosis, sympathetic cardiac innervation seems to play an important role in the pathogenesis of AF [8,9]. Cardiac single-photon emission computed tomography (SPECT) imaging using the radiotracer ^123^I-metaiodobenzylguanidine (^123^I-mIBG) has been successfully employed to assess cardiac sympathetic nervous function in various clinical scenarios [10-12]. Data from small clinical studies suggest that ^123^I-mIBG imaging in patients with paroxysmal AF could support clinical risk stratification. In a recent study, a reduced H/M ratio for ^123^I-mIBG has been identified as a predictor for the development of permanent AF and heart failure associated with permanent AF [8]. Arimoto et al. demonstrated that a high global washout rate (WR) of ^123^I-mIBG (5 days after PVI) is an independent predictor of AF relapses following PVI in patients with either paroxysmal or permanent AF [9]. As a potential limitation, these studies analyzed the global parameters from planar imaging (H/M ratio and WR) only. Therefore, we hypothesized that PVI is not only associated with global but also with regional changes of the cardiac sympathetic activity. Here, we describe a prospective series of 16 patients with paroxysmal AF who underwent serial ^123^I-mIBG SPECT/CT imaging before and 4 weeks after PVI and were followed up for AF relapses.

## Methods

### Patients

We included 16 consecutive patients (11 men, 5 women) with paroxysmal AF referred to our clinic for PVI who received serial ^123^I-mIBG SPECT/CT. Patients were between 40 and 72 years old and received standard antiarrhythmic therapy including beta blockers, flecainide, and dronedarone which was mostly continued after PVI as a prophylactic therapy (patients characteristics, Table 1). Except the two individuals, no history of coronary artery disease was known. These two individuals had no history of myocardial infarction, had normal left ventricular ejection fraction (>55%), and stress-induced myocardial ischemia had been excluded by myocardial perfusion SPECT/CT (one patient) or coronary artery angiography (one patient). The local institutional ethical committee on human research approved the evaluation and presentation of the patient data. Informed consent was given by the patients.

**Table 1:** Individual patients characteristics

**Individual number**	**Sex**	**Age**	**Duration of AF (years)**	**CHD**	**LA volume (ml)**	**AA drug treatment before PVI**	**Duration of radiofrequency impulses (seconds)**	**Relapse**	**AA Drug treatment after PVI**
1	F	51	2	One vessel CHD	99	Sotalol	56	Yes, re-PVI	Sotalol
2	M	55	10	No CHD	122	Beta blocker	41	Yes, re-PVI	Beta blocker
3	F	67	7	No CHD	123	Beta blocker, dronedarone	61	Yes, re-PVI	Beta blocker, dronedarone
4	M	44	13	No CHD	72	Beta blocker	40	None	Beta blocker, flecainide
5	F	56	4	No CHD	118	Beta blocker, flecainide	79	None	Beta blocker, flecainide
6	M	54	1	No CHD	76	Beta blocker, flecainide	61	None	Beta blocker, flecainide
7	M	72	17	No CHD	82	Beta blocker, dronedarone	69	None	Beta blocker, dronedarone
8	M	69	3	three vessel CHD	183	Beta blocker	98	Yes, re-PVI	Beta blocker, dronedarone
9	M	64	2	No CHD	125	Beta blocker, flecainide	91	None	Beta blocker, propafenone
10	F	49	5	No CHD	82	Beta blocker, flecainide	81	None	Beta blocker, flecainide
11	M	48	1	No CHD	116	Beta blocker, dronedarone	43	None	Dronedarone
12	F	70	2	No CHD	N/A	Beta blocker, dronedarone	40	Yes, no re-PVI	Beta blocker, flecainide
13	M	52	5	No CHD	103	Beta blocker, flecainide	33	None	Beta blocker, flecainide
14	M	40	1	No CHD	52	Beta blocker	41	None	Beta blocker, flecainide
15	M	61	4	No CHD	115	Beta blocker, flecainide	49	None	Beta blocker, flecainide
16	M	52	5	No CHD	103	Beta blocker, flecainide	51	None	Beta blocker, flecainide

CHD, chronic ischemic heart disease; LA, left atrium; AA, antiarrhythmic, N/A not applicable.

### Radionuclide (^123^I-mIBG) imaging

According to a proposal for standardization of ^123^I-mIBG cardiac sympathetic imaging by the EANM Cardiovascular Committee and the European Council of Nuclear Cardiology [13], all patients received 370 MBq of ^123^I-mIBG (AdreView, GE Healthcare, Little Chalfont, Buckinghamshire, UK) and underwent anterior planar imaging of the thorax SPECT/CT imaging of the heart at 15 min and 4 h post injection. Data acquisition was performed on a hybrid two-slice SPECT/CT device (Symbia T2, Siemens Medical Solutions, Malvern, PA, USA). Emission data were acquired with parallel-hole, medium-energy, high-resolution collimators, the two detector heads positioned in a 90° angle, with a 20% symmetric energy window centered at 159 keV. Further acquisition parameters were 32 rotation steps, with 2.8° rotation per stop, 90° each head, and 25 s per rotational projection. All SPECT data were acquired electrocardiogram (ECG)-gated (eight ECG gates). The left ventricular end-systolic and end-diastolic volumes (ESV, EDV) and left ventricular ejection fraction (LVEF) were automatically calculated from the ECG-gated SPECT data of the late (4 h p.i.) study by means of the Corridor 4D-MSPECT software package (version 5.1, INVIA Medical Imaging Solutions, Ann Arbor, MI, USA). All SPECT data were corrected for attenuation by CT-attenuation correction through a low-dose spiral CT scan performed directly after acquisition of the emission data. CT scans were acquired during free breathing without ECG-gating, at spiral mode with pitch 1.4, tube voltage of 130 kV, 30 mAs, scan time 14.67 s, CTDI (computed tomography dose index) of approximately 3.2, and DLP (dose-length product) of 55. The CT images were reconstructed at a 5.0-mm thickness by using a reconstruction algorithm with a 512 × 512 matrix and a full-chest-size-adapted FOV of 50 × 50 cm^2^.

### Analysis

#### Analysis of planar images

The H/M ratio was determined from the counts/pixel in a visually drawn heart region of interest (ROI) divided by the counts/pixel in a visually drawn mediastinum ROI in the midline upper chest positioned to reflect the region with lowest background activity, for the early images as well as for the late images. The mediastinal ROI had a standardized size of 1,200 mm^2^. The global WR was calculated as described elsewhere [13].

#### SPECT analysis

For *semiquantitative SPECT image analysis*, only attenuation-corrected early/late images were analyzed by using the Corridor 4D-MSPECT software package (version 5.1, INVIA Medical Imaging Solutions, Ann Arbor, MI, USA). Images were resliced into short and horizontal/vertical long axis slices for clinical reading. Additionally, bull's eye plots and a 17-segment model of the left ventricle were calculated for each SPECT images set. All images were interpreted by two experienced and independent nuclear cardiologists blinded to the clinical data. Each reader scored all SPECT image sets: relative regional tracer uptake in relation to the maximum regional myocardial ^123^I-mIBG uptake was classified using a 17-segment model of the left ventricle and a semiquantitative five-point scale (0 = normal uptake, 1 = mildly reduced uptake, 2 = moderately reduced uptake, 3 = severely reduced uptake and 4 = absent uptake), as previously described [14-16]. A ‘summed defect score’ (SDS) was calculated as the sum of all segmental defect scores in the late study (4 h p.i.). A SDS ≥ 3 was considered to be an innervation deficit. In case a single abnormal segment showed a defect score of 1 or 2, at least two abnormal segments had to be adjacent to each other to account for an SDS ≥ 3. Segments showing a score of 3 or 4 did not necessarily need to be adjacent. For *quantitative SPECT analysis*, a 17-segment model of the left ventricle was calculated by means of a contour finding algorithm, developed and validated by our group and previously published (ESM, elastic surface model [17]). Left ventricular segmentation was performed automatically. Segmental ^123^I-mIBG washout was calculated as the difference of the percentage ^123^I-mIBG uptakes between the early (15 min p.i.) and late (4 h p.i.) SPECT images. Segmental left ventricular ^123^I-mIBG uptake and washout (before PVI vs. after PVI) were compared statistically.

### Computed tomography

CT angiography of the atria and pulmonary veins was performed in 15 out of 16 patients for preinterventional of PVI. All CT examinations were acquired using dual-source CT systems (either Somatom Definiton or Somatom Definiton Flash, Siemens Medical Solutions, Malvern, PA, USA). Examinations were performed at 100 kV according to a non-ECG-triggered high-pitch (3,2) protocol. Collimation was 64 × 0.6 mm^2^ for the Somatom Definition and 128 × 0.6 mm^2^ for the Somatom Definition Flash. A total of 60 ml of non-ionic, iodinated contrast agent (Ultravist 370, Bayer-Schering-Pharma, Berlin, Germany) was injected through a right antecubital vein at a flow rate of 5 ml/s, followed by a 50-ml saline chaser bolus at the same flow rate. Data acquisition was initiated by a bolus-triggering technique (CARE Bolus, Siemens Medical Solutions) with the region of interest placed within the left atrium. The threshold above the native reference scan and the delay before scanning started were 100 HU respectively 6 s for the Somatom Definition and 150 HU respective 10 s for the Somatom Definition Flash. Measurements of the left atrial size were performed on transverse 1-mm slices with a reconstruction increment of 1 mm using commercially available software (SyngoVolumetry, Siemens Medical Systems, Malvern, PA, USA). If ECG-trigger was available (1 case), a diastolic reconstruction interval was chosen by ECG; otherwise, the diastole (11 cases) was identified by evaluation of the valve's status. The pulmonary veins were excluded from the atrial volume.

### Pulmonary vein isolation

PVI was carried out as described previously [3,18]. All patients signed written informed consent and transesophageal echocardiography was performed in all patients prior to the procedure. The ablation was performed in the fasting state under sedation with midazolam and pain medication with piritramide as needed. Surface ECG and endocardial electrograms were continuously monitored on a computer-based digital amplifier/recorder system (Siemens Axiom Sensis XP or Prucka, GE Medical Systems Inc., Milwaukee, WI, USA). Two 6-F sheaths were placed in the left groin. A decapolar steerable catheter (St. Jude Medical, Inc., St. Paul, MN, or Inquiry, IBI, Irvine Biomedical, Inc., Irvine, CA, USA) was placed in the coronary sinus via one of the sheaths. Two 8-F sheaths were placed in the right groin, one being an SL1-sheath (Daig SL1, St. Jude Medical, Inc., Little Canada, MN, USA) for transseptal puncture. Selective angiography of the PV was performed with about 40 ml of non-ionic contrast (Ultravist 370, Bayer, Berlin, Germany), in posterior-anterior and left anterior oblique 60° projections. The 3D geometry of the left atrium was acquired using the Ensite NavX Velocity (St. Jude Medical) or the CARTO System (Biosense Webster, Diamond Bar, CA, USA) or the NaVX-Fusion System (St. Jude Medical, Inc.). The acquired geometry was fused with the left atrial 3D reconstruction of the computed tomographic (CT) data, previously performed with the Ensite Verismo software. Thereafter, an antral point-by-point circumferential ablation around the ipsilateral PV, with a distance of 0.5 to 1.0 cm from the ostia, using a 4-mm open-tip irrigated catheter (IBI Therapy Coolpath Duo 7-Fr, St. Jude Medical), was performed. The maximum power was set to 30 W, going selectively up to 40 W if the PV isolation could not be achieved, especially at the anterior ridge border of the lateral PVs. Temperature was limited to 43°C. Irrigation was adjusted manually between 17 and 30 ml/min. The complete electrical isolation was monitored and confirmed by the decapolar catheter during sinus rhythm and differential pacing maneuvers.

### Statistics

Values are expressed as mean ± standard deviation. Differences in mean values were compared by either a Wilcoxon test or a Mann–Whitney *U* test for unpaired samples. A *p* < 0.05 indicated a statistically significant difference. All statistical analyses were performed using a commercially available software package (SPSS, version 17.0, SPSS Inc., Chicago IL, USA).

## Results

### Cardiac sympathetic activity before and after PVI

#### Analysis of global sympathetic cardiac innervation

**Figure 1:**
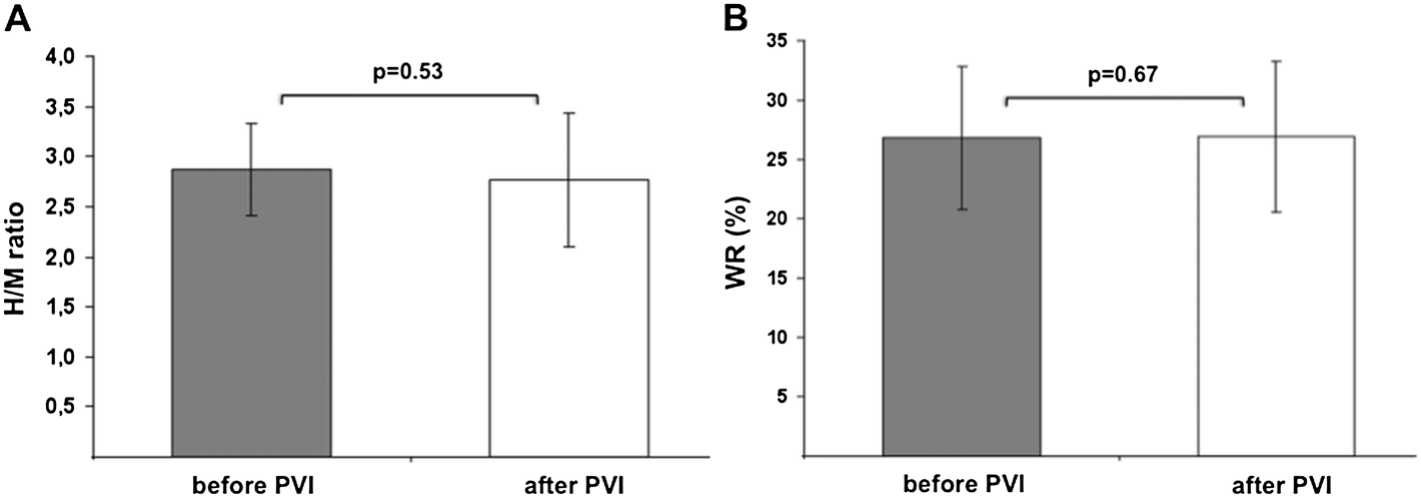
**Mean late H/M ratios and global WRs.** Late H/M ratios **(A)** and global WR **(B)** before and after PVI indicate no changes in global sympathetic innervation.

Mean late H/M ratios and global WRs did not change from baseline to the follow-up scan after PVI (pre, 2.9 ± 0.5 vs. post, 2.7 ± 0.6, *p* = 0.53 and pre, 26.8% ± 6.0% vs. post, 26.9% ± 3.6%, *p* = 0.67, respectively; Figure 1A,B).

#### Analysis of regional sympathetic cardiac innervation

**Figure 2:**
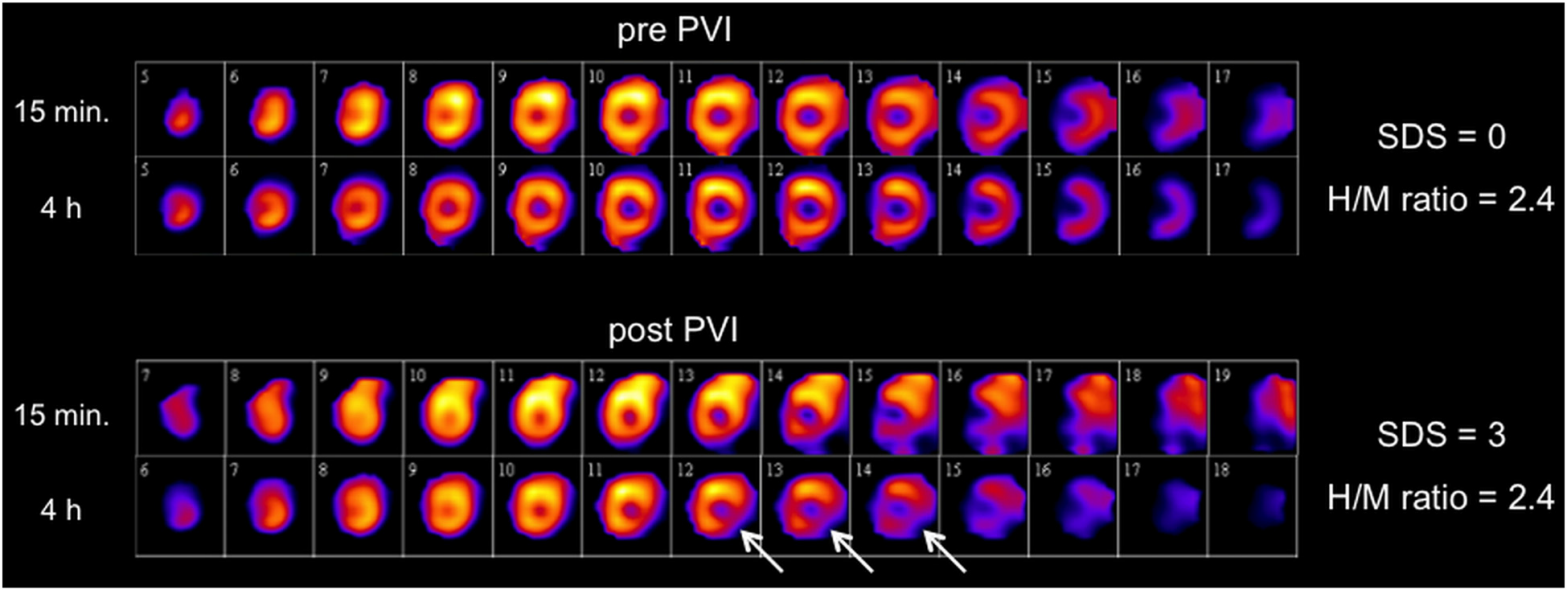
**Example of a patient with a new innervation defect after PVI.** Short axis slices of the left ventricle 15 min and 4 h after ^123^I-mIBG injection before and after PVI. Regional late innervation deficit/increased regional washout after PVI affecting the basal inferolateral wall (white arrow). Increasing SDS (summed defect score) but Stable H/M ratio.

**Figure 3:**
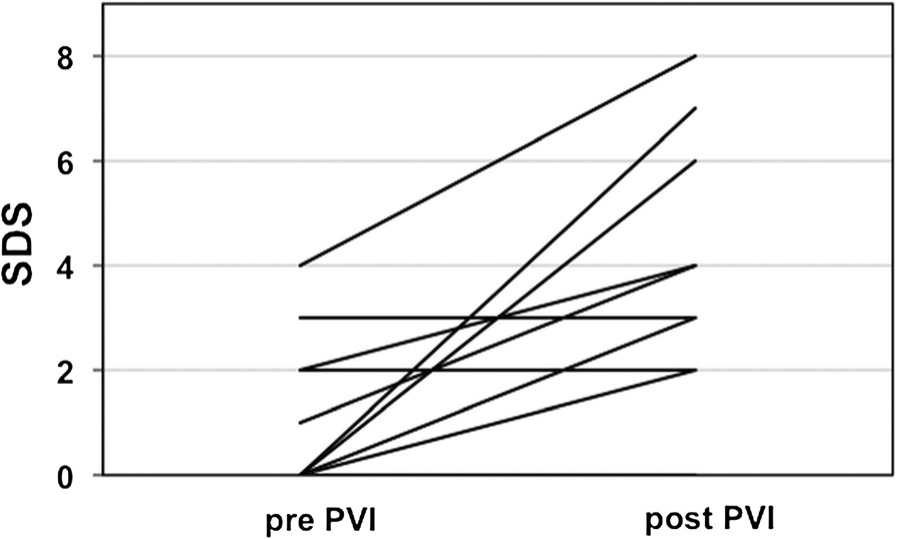
Development of the SDS (summed defect score) in the individual patients (pre-PVI versus post PVI).

**Figure 4:**
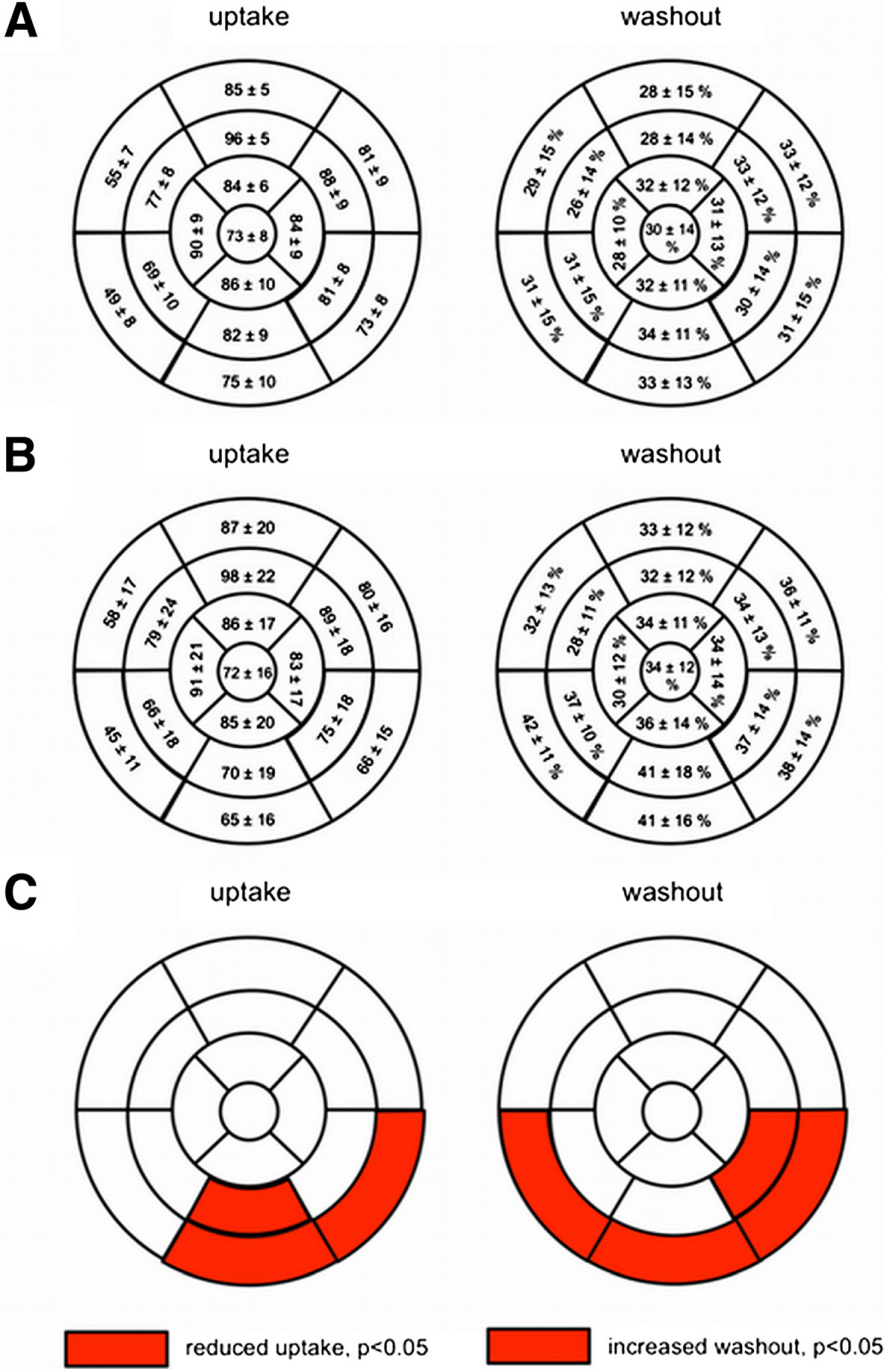
**Quantitative 17-segmental analysis.** Quantitative 17-segmental analysis of the late ^123^I-mIBG uptake (left) and washout (right) of the left ventricle before **(A)** and after **(B)** PVI. Values are expressed as mean regional uptake in comparison to the regional maximum uptake and segmental washout (%). Significantly reduced uptake and increased regional washout after PVI (mean values after PVI compared with mean values before PVI) in the basal inferior and basal lateral segments **(C)**.

At baseline, no regional defects were observed in the early (15 min p.i.), except in four patients (25%) in the late (4 h p.i.) SPECT images. These defects were located in the inferolateral (three patients) or the lateral wall (one patient). After PVI, regional deficits were present in two patients in the early but in the ten patients (62.5%) in the late images including the four patients with preexisting deficits which remained unchanged. Newly emerging defects post PVI were localized in the inferolateral (four patients) and the apical or basal posterior wall (one patient each), respectively (patient example, Figure 2). Semiquantitative analysis of all subjects showed a significant increase in the mean summed defect score (4 h p.i.) from baseline to post PVI (pre, 0.8 ± 1.3 vs. post, 2.6 ± 2.7, *p* = 0.011; Figure 3). The quantitative SPECT analysis comparing the late (4 h p.i.) ^123^I-mIBG uptake before and after PVI showed a significant reduction of uptake in the left ventricular segments 4, 5, and 10 and a significantly increased regional washout in the segments 3, 4, 5, and 11 (Figure 4). In contrast, H/M ratios did not differ significantly between patients with (*n* = 10) and patients without regional defects (*n* = 6) after PVI (2.8 ± 0.6 vs. 2.7 ± 0.7, *p* = not significant (ns)). Accordingly, global WRs were similar between these both groups (26.8% ± 5.0% vs. 27.8% ± 7.2%, *p* = ns).

### Innervation defects and AF relapses

**Figure 5:**
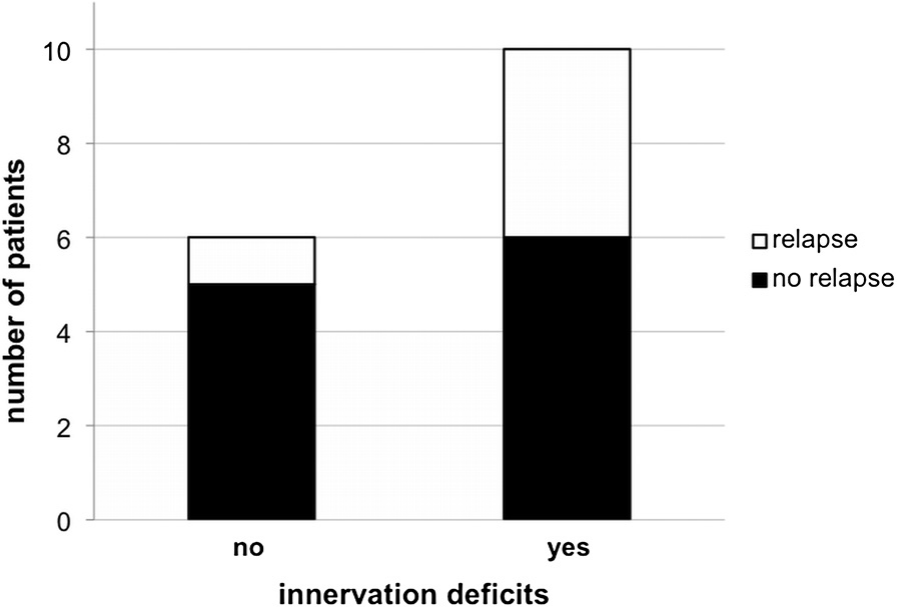
Number of relapses in patients without (1/6) and with (4/10) innervation deficits post PVI.

In the short-term follow-up period of 6 months, four out of ten patients (40%) with regional innervation defects after PVI suffered from an early recurrence of AF. In contrast, only one of the six patients (17%) without detectable regional defects of myocardial ^123^I-mIBG uptake after PVI had a clinically apparent early relapse of AF (Figure 5). In case of the four patients with preexisting and persisting defects, one patient (25%) suffered from a clinical relapse. However, the mean SDS after PVI were slightly but not significantly higher in patients with and AF relapses compared with patients without relapses (no relapse *n* = 11:2.4 ± 1.5 vs. relapse *n* = 4:2.7 ± 3.1; *p* = ns). The formally calculated sensitivity, specificity, positive predictive value and negative predictive value were 80%, 45%, 40%, and 83%.

### Left atrial volumes and AF relapse

Except one patient, left atrial volumes were calculated on the basis of contrast enhanced CT data. Patients suffering from a relapse showed a tendency towards larger left atrial volumes [95 ± 23 ml vs. 132 ± 36 ml (no relapse vs. relapse; *n* = 11 vs. 4)]. However, statistical analysis revealed no significant differences (*p* = ns).

### Left ventricular function and AF relapse

Left ventricular volumes (ESV, EDV) and LVEF were preserved in every patient and did not change significantly after PVI (ESV, pre 36 ± 14 ml vs. post 35 ± 15 ml; EDV, pre 107 ± 24 ml vs. post 102 ± 28 ml; LVEF, pre 66% ± 7% vs. post 67% ± 9%; all *p* = ns). These parameters did not differ between patients without or with AF relapse either [ESV, 37 ± 10 ml vs. 33 ± 20 ml; EDV, 106 ± 19 ml vs. 108 ± 44 ml; LVEF, 65% ± 6% vs. 70% ± 7%; all *p* = ns (no relapse vs. relapse; *n* = 11 vs. 4)].

## Discussion

### PVI causes changes in cardiac sympathetic innervation

Solid evidence supports the hypothesis that the sympathetic cardiac nervous system plays a key role in the initiation and propagation of cardiac arrhythmias including paroxysmal AF [19-22]. Data from animal studies suggest that sympathetic innervation changes lead to alterations rendering the atrium more vulnerable to the arrhythmogenic triggers originating from the pulmonary veins [23-26]. Recently, Akutsu et al. performed a cardiac ^123^I-mIBG study in patients with paroxysmal AF [8]. They found that a reduced H/M ratio was a powerful independent predictor of the development of permanent AF and heart failure plus permanent AF. Arimoto et al. were the first to perform cardiac ^123^I-mIBG imaging in patients with AF after treatment with PVI [9]. In contrast to our data, they reported a high WR as an independent predictor of AF recurrence in patients with paroxysmal or permanent AF, a finding which is possibly missed by our study due to the size of the patient cohort. On the contrary, a subgroup of 20 patients underwent ^123^I-mIBG imaging before and after PVI. In this group neither the H/M ratio nor the global WR changed significantly after treatment. This finding is in line with our results, but in contrast to the study by Arimoto et al. [9], we additionally assessed segmental tracer uptake known to improve the sensitivity and specificity of the imaging technique [27]. Hence, we observed regionally increased washout and reduced late segmental ^123^I-mIBG uptake in 25% of the patients already before PVI and newly emerging innervation defects in 37.5% of patients after PVI.

### Changes in sympathetic innervation are located in the posterolateral wall

^123^I-mIBG SPECT/CT analysis shows that the regional defects are located in the inferior lateral wall of the left ventricle, both before and after PVI, but more frequently after PVI. Interestingly, while atrial ganglionated plexuses seem to be located in multiple locations on atrial chamber walls [21], different studies suggest that ventricular ganglionated plexuses appear to be primarily associated with the origins of major cardiac blood vessels. In addition, there is a high density of nerves near the venous ostia and along the pulmonary veins. Various anatomical studies (reviewed in [21]) implicate that these nerves appear to originate in the ganglionated plexuses along the middle and dorsum of the right atrium, while the corresponding nerve endings seem to penetrate mostly at the root of the veins.

As a firstly intriguing explanation, the observed innervation defects could be interpreted as a consequence of direct denervation due to ablation of sympathetic nerve fibers around the pulmonary veins similar to the results of a study performed by Mabucchi et al. [28]. These authors described a partial left ventricular sympathetic denervation assessed by cardiac ^123^I-mIBG imaging after mitral valve surgery. Alternatively, the degree of rhythm control following PVI might offer another interpretation of our results. In this model, the interplay between sinus rhythm and atrial fibrillation episodes alters sympathetic innervation and therefore leads to regional innervation imbalances. More specifically, the occurrence of paroxysmal AF with short duration may cause the appearance of regional denervation. Hence, ventricular innervation imbalances may reflect primarily atrial innervation imbalances.

### Regional changes in sympathetic innervation affect both uptake and washout

The results from our study show convincingly that the changes in sympathetic innervation affect both uptake and washout. A number of studies dealing with cardiac ^123^I-mIBG imaging in several cardiac diseases have noted decreased global cardiac ^123^I-mIBG uptake (H/M ratio) in combination with increased global washout. In a variety of different conditions such as congestive heart failure, coronary artery disease, and inherited arrhythmias, such changes have been demonstrated to indicate disease progression or an adverse prognosis [8,29-35]. Additionally, pharmaceutical and device therapy in some of these conditions has resulted in recession of these findings [36-38]. The fact that different cardiac diseases are associated with the changes in cardiac uptake or washout of ^123^I-mIBG suggests the presence of a common reaction of the cardiac sympathetic nervous system [39]. It has been suggested that one such mechanism could be a high rate of post-ganglionic cardiac sympathetic traffic whereby the locally increased rate of translocation of neurotransmitter vesicles to the membrane surface and an increased rate of exocytosis result in decreased regional ^123^I-mIBG uptake and higher washout. The results of our study point to the development of regional imbalances of sympathetic innervation with either increased regional sympathetic tone (reflected by the increased regional washout) or innervation defects in patients with PAF (paroxysmal atrial fibrillation) both before and after PVI.

Since sympathetic innervation is considered to be a highly dynamic process with an ability to self-regenerate [28], the findings suggest that the cardiac sympathetic nervous system might play a role both in the remodeling process that is associated with paroxysmal AF and the reverse remodeling following PVI. Thus, the remodeling process of the sympathetic nervous system might have an impact on the likelihood to develop an AF relapse in the short-term course following PVI. Clinical studies indicate that this period of time is crucial for the atrial reverse remodeling process that determines the probability of sinus rhythm maintenance [2,40-42]. In patients with more intense atrial remodeling the remodeling process may result in a more extensive sympathetic damage and subsequently to more severe left ventricular denervation.

### Regional sympathetic innervation deficits might help identify patients at risk for AF relapse

Hence, we hypothesize that regional defects of cardiac ^123^I-mIBG uptake and increased regional washout are hallmarks of sympathetic innervation imbalances, which may be useful parameters for the non-invasive risk stratification of AF occurrences. Although based on findings in a relatively small patient cohort the results of our study suggest a certain prognostic value of ^123^I-mIBG SPECT/CT. As the absence of innervation deficits was indicative for a better outcome with respect to the occurrence of AF relapses, the negative predictive value of ^123^I-mIBG SPECT/CT may be high, a preliminary finding which has to be interpreted with caution and has to be proved in a larger patient cohort. Apart from that, patients with larger left atrial volumes tend to have more AF relapses following PVI [40,41]. Accordingly, patients with AF relapses in our study tended to have larger left atrial volumes. However, the differences in atrial size in comparison to patients without AF relapse did not reach the level of statistical significance.

Left ventricular function was preserved in our patient cohort, did not change after PVI, and did not correlate with AF relapses. Therefore, our imaging results should not be confounded by the presence of heart failure. Moreover, relevant coronary artery disease was excluded in our patient cohort.

Further studies with larger patient cohorts are needed to clarify the value of ^123^I-mIBG SPECT/CT for risk stratification and adequate patient selection of AF patients for PVI. It remains to be determined whether regional sympathetic innervation deficits are a surrogate parameter for incomplete PVI with respect to the middle and/or long-term outcome. In addition, further research is necessary to decipher the effect of cardiac sympathetic innervation inhomogeneity on susceptibility to AF in patients suffering from AF paroxysms and on AF relapses in patients following PVI.

### Limitations

This observational study does not contain data from a control group treated without PVI, since we compare cardiac innervation status before PVI with that after PVI. Beyond patient-related issues, the imaging method chosen in this clinical study allows for ventricular imaging only, since due to the low mass of the atria only left ventricular innervation can be assessed and local sympathetic innervation changes around the pulmonary veins could not be adequately measured.

Measurements of left atrial volumes are not exact since the majority of CT scans were performed non-ECG-triggered.

## Conclusions

A significant number of patients with paroxysmal AF show regional sympathetic innervation deficits already before PVI. In addition, PVI is obviously associated with newly emerging regional cardiac innervation defects mainly observed in the inferior lateral wall. However, it is yet unclear whether these regional changes in the cardiac sympathetic innervation arise from an atrophy of sympathetic nerve plexus due to PVI or whether they can be attributed to a significant reduction of AF burden after PVI. Moreover, although in a small set of patients, the absence of innervation defects may be an indicator for a better outcome and the presence of regional innervation defects after PVI may be indicative for the risk of AF relapses. In summary, cardiac sympathetic innervation seems to play a role in AF and PVI. Larger prospective studies are needed to clarify these issues.

## Abbreviations

AA: Antiarrhythmic; AF: Atrial fibrillation; CHD: Chronic ischemic heart disease; CT: Computed tomography; EDV: End-diastolic volume; ESV: End-systolic volume; H/M ratio: Heart-to-mediastinum ratio; HU: Hounsfield units; I-mIBG: Iodine-123-meta-iodobenzylguanidine; LA: Left atrium; LVEF: Left ventricular ejection fraction; PVI: Pulmonary vein isolation; ROI: Region of interest; SDS: Summed defect score; SPECT/CT: Single-photon emission computed tomography with low-dose computed tomography for attenuation correction; WR: Washout-rate.

## Competing interests

The authors declare that they have no competing interests.

## Authors’ contributions

CW and PL analyzed the SPECT/CT data and drafted the manuscript. CW and AV performed the SPECT/CT scan. PL carried out the patient's follow-up; CS performed and analyzed CT angiography. GM was responsible for patient recruitment and LS performed pulmonary vein isolation. MS and LS conceived of the study. OS, LS, and MS participated in the design of the study and drafted the manuscript. All authors read and approved the final manuscript.

## Authors’ information

CW, AV, OS, and MS are specialist and scientists in nuclear medicine specializing in nuclear cardiology. PL, GM, and LE are cardiologists specializing in cardiac electrophysiology. CS is radiologist and specialist in cardiac imaging.

## References

[B1] Wasmer K Eckardt L Management of atrial fibrillation around the world: a comparison of current ACCF/AHA/HRS, CCS, and ESC guidelines Europace 2011 3 1368 1374 10.1093/europace/eur172 21712281

[B2] Wazni OM Fahmy TS Natale A Circumferential pulmonary-vein ablation for atrial fibrillation N Engl J Med 2006 3 2289 2291 16723624 10.1056/NEJMc060884

[B3] Wasmer K Monnig G Bittner A Dechering D Zellerhoff S Milberg P Kobe J Eckardt L Incidence, characteristics, and outcome of left atrial tachycardias after circumferential antral ablation of atrial fibrillation Heart Rhythm 2012 3 1660 1666 10.1016/j.hrthm.2012.06.007 22683745

[B4] Akoum N McGann C Vergara G Badger T Ranjan R Mahnkopf C Kholmovski E Macleod R Marrouche N Atrial fibrosis quantified using late gadolinium enhancement MRI is associated with sinus node dysfunction requiring pacemaker implant J Cardiovasc Electrophysiol 2012 3 44 50 10.1111/j.1540-8167.2011.02140.x 21806700 PMC4465539

[B5] Badger TJ Adjei-Poku YA Marrouche NF MRI in cardiac electrophysiology: the emerging role of delayed-enhancement MRI in atrial fibrillation ablation Future Cardiol 2009 3 63 70 10.2217/14796678.5.1.63 19371204

[B6] Daccarett M McGann CJ Akoum NW MacLeod RS Marrouche NF MRI of the left atrium: predicting clinical outcomes in patients with atrial fibrillation Expert Rev Cardiovasc Ther 2011 3 105 111 10.1586/erc.10.177 21166532 PMC3121930

[B7] Vergara GR Marrouche NF Tailored management of atrial fibrillation using a LGE-MRI based model: from the clinic to the electrophysiology laboratory J Cardiovasc Electrophysiol 2011 3 4 481 487 10.1111/j.1540-8167.2010.01941.x 21044212

[B8] Akutsu Y Kaneko K Kodama Y Li HL Suyama J Shinozuka A Gokan T Hamazaki Y Tanno K Kobayashi Y Iodine-123 mIBG imaging for predicting the development of atrial fibrillation JACC Cardiovasc Imaging 2011 3 78 86 10.1016/j.jcmg.2010.10.005 21232708

[B9] Arimoto T Tada H Igarashi M Sekiguchi Y Sato A Koyama T Yamasaki H Machino T Kuroki K Kuga K Aonuma K High washout rate of iodine-123-metaiodobenzylguanidine imaging predicts the outcome of catheter ablation of atrial fibrillation J Cardiovasc Electrophysiol 2011 3 1297 1304 10.1111/j.1540-8167.2011.02123.x 21692898

[B10] Arimoto T Takeishi Y Niizeki T Nozaki N Hirono O Watanabe T Nitobe J Tsunoda Y Suzuki S Koyama Y Kitahara T Okada A Takahashi K Kubota I Cardiac sympathetic denervation and ongoing myocardial damage for prognosis in early stages of heart failure J Card Fail 2007 3 34 41 10.1016/j.cardfail.2006.09.002 17339001

[B11] Ji SY Travin MI Radionuclide imaging of cardiac autonomic innervation J Nucl Cardiol 2010 3 655 666 10.1007/s12350-010-9239-x 20454877

[B12] Merlet P Pouillart F Dubois-Rande JL Delahaye N Fumey R Castaigne A Syrota A Sympathetic nerve alterations assessed with 123I-MIBG in the failing human heart J Nucl Med 1999 3 224 231 10025827

[B13] Flotats A Carrio I Agostini D Le Guludec D Marcassa C Schafers M Somsen GA Unlu M Verberne HJ Proposal for standardization of 123I-metaiodobenzylguanidine (MIBG) cardiac sympathetic imaging by the EANM Cardiovascular Committee and the European Council of Nuclear Cardiology Eur J Nucl Med Mol Imaging 2010 3 1802 1812 10.1007/s00259-010-1491-4 20577740

[B14] Cerqueira MD Weissman NJ Dilsizian V Jacobs AK Kaul S Laskey WK Pennell DJ Rumberger JA Ryan T Verani MS Standardized myocardial segmentation and nomenclature for tomographic imaging of the heart. A statement for healthcare professionals from the Cardiac Imaging Committee of the Council on Clinical Cardiology of the American Heart Association Int J Cardiovasc Imaging 2002 3 539 542 12135124

[B15] Germano G Kavanagh PB Waechter P Areeda J Van Kriekinge S Sharir T Lewin HC Berman DS A new algorithm for the quantitation of myocardial perfusion SPECT. I: technical principles and reproducibility J Nucl Med 2000 3 712 719 10768574

[B16] Sharir T Germano G Waechter PB Kavanagh PB Areeda JS Gerlach J Kang X Lewin HC Berman DS A new algorithm for the quantitation of myocardial perfusion SPECT. II: validation and diagnostic yield J Nucl Med 2000 3 720 727 10768575

[B17] Stegger L Lipke CS Kies P Nowak B Schober O Buell U Schafers M Schaefer WM Quantification of left ventricular volumes and ejection fraction from gated 99mTc-MIBI SPECT: validation of an elastic surface model approach in comparison to cardiac magnetic resonance imaging, 4D-MSPECT and QGS Eur J Nucl Med Mol Imaging 2007 3 900 909 10.1007/s00259-006-0322-0 17216166

[B18] Bittner A Monnig G Zellerhoff S Pott C Kobe J Dechering D Milberg P Wasmer K Eckardt L Randomized study comparing duty-cycled bipolar and unipolar radiofrequency with point-by-point ablation in pulmonary vein isolation Heart Rhythm 2011 3 1383 1390 10.1016/j.hrthm.2011.03.051 21457793

[B19] Chen PS Tan AY Autonomic nerve activity and atrial fibrillation Heart Rhythm 2007 3 3 Suppl S61 S64 17336887 10.1016/j.hrthm.2006.12.006 PMC1852524

[B20] Crawford MH Does cardiac sympathetic innervation imaging fulfill an unmet need for managing atrial fibrillation? JACC Cardiovasc Imaging 2011 3 87 88 10.1016/j.jcmg.2010.11.008 21232709

[B21] Kapa S Venkatachalam KL Asirvatham SJ The autonomic nervous system in cardiac electrophysiology: an elegant interaction and emerging concepts Cardiol Rev 2010 3 275 284 10.1097/CRD.0b013e3181ebb152 20926936

[B22] Nakagawa H Scherlag BJ Patterson E Ikeda A Lockwood D Jackman WM Pathophysiologic basis of autonomic ganglionated plexus ablation in patients with atrial fibrillation Heart Rhythm 2009 3 12 Suppl S26 S34 19959140 10.1016/j.hrthm.2009.07.029

[B23] Chang CM Wu TJ Zhou S Doshi RN Lee MH Ohara T Fishbein MC Karagueuzian HS Chen PS Chen LS Nerve sprouting and sympathetic hyperinnervation in a canine model of atrial fibrillation produced by prolonged right atrial pacing Circulation 2001 3 22 25 10.1161/01.CIR.103.1.22 11136680

[B24] Saygili E Schauerte P Kuppers F Heck L Weis J Weber C Schwinger RH Hoffmann R Schroder JW Marx N Rana OR Electrical stimulation of sympathetic neurons induces autocrine/paracrine effects of NGF mediated by TrkA J Mol Cell Cardiol 2010 3 79 87 10.1016/j.yjmcc.2010.01.019 20138055

[B25] Swissa M Zhou S Paz O Fishbein MC Chen LS Chen PS Canine model of paroxysmal atrial fibrillation and paroxysmal atrial tachycardia Am J Physiol 2005 3 H1851 H1857 10.1152/ajpheart.00083.2005 16006551

[B26] Waldo AL Mechanisms of atrial fibrillation J Cardiovasc Electrophysiol 2003 3 12 Suppl S267 S274 15005213 10.1046/j.1540-8167.2003.90401.x

[B27] Chirumamilla A Travin MI Cardiac applications of 123I-mIBG imaging Sem Nucl Med 2011 3 374 387 10.1053/j.semnuclmed.2011.04.001 21803188

[B28] Mabuchi M Imamura M Kubo N Morita K Noriyasu K Tsukamoto T Yasuda K Tamaki N Sympathetic denervation and reinnervation after the maze procedure J Nucl Med 2005 3 1089 1094 16000276

[B29] Akutsu Y Kaneko K Kodama Y Li HL Kawamura M Asano T Tanno K Shinozuka A Gokan T Kobayashi Y Cardiac sympathetic nerve abnormality predicts ventricular tachyarrhythmic events in patients without conventional risk of sudden death Eur J Nucl Med Mol Imaging 2008 3 2066 2073 10.1007/s00259-008-0879-x 18622611

[B30] Akutsu Y Kaneko K Kodama Y Li HL Kawamura M Asano T Tanno K Shinozuka A Gokan T Kobayashi Y The significance of cardiac sympathetic nervous system abnormality in the long-term prognosis of patients with a history of ventricular tachyarrhythmia J Nucl Med 2009 3 61 67 19091900 10.2967/jnumed.108.055194

[B31] Bax JJ Kraft O Buxton AE Fjeld JG Parizek P Agostini D Knuuti J Flotats A Arrighi J Muxi A Alibelli MJ Banerjee G Jacobson AF 123 I-mIBG scintigraphy to predict inducibility of ventricular arrhythmias on cardiac electrophysiology testing: a prospective multicenter pilot study Circulation 2008 3 131 140 19808530 10.1161/CIRCIMAGING.108.782433

[B32] Boogers MJ Borleffs CJ Henneman MM van Bommel RJ van Ramshorst J Boersma E Dibbets-Schneider P Stokkel MP van der Wall EE Schalij MJ Bax JJ Cardiac sympathetic denervation assessed with 123-iodine metaiodobenzylguanidine imaging predicts ventricular arrhythmias in implantable cardioverter-defibrillator patients J Am Coll Cardiol 2011 3 2769 2777 10.1016/j.jacc.2009.12.066 20538172

[B33] Carrio I Cowie MR Yamazaki J Udelson J Camici PG Cardiac sympathetic imaging with mIBG in heart failure JACC Cardiovasc Imaging 2010 3 92 100 10.1016/j.jcmg.2009.07.014 20129538

[B34] Marshall A Cheetham A George RS Mason M Kelion AD Cardiac iodine-123 metaiodobenzylguanidine imaging predicts ventricular arrhythmia in heart failure patients receiving an implantable cardioverter-defibrillator for primary prevention Heart 2012 3 1359 1365 10.1136/heartjnl-2012-302321 22904144

[B35] Paul M Wichter T Kies P Gerss J Wollmann C Rahbar K Eckardt L Breithardt G Schober O Schulze-Bahr E Schafers M Cardiac sympathetic dysfunction in genotyped patients with arrhythmogenic right ventricular cardiomyopathy and risk of recurrent ventricular tachyarrhythmias J Nucl Med 2011 3 1559 1565 10.2967/jnumed.111.088997 21908389

[B36] Burri H Sunthorn H Somsen A Fleury E Stettler C Shah D Righetti A Improvement in cardiac sympathetic nerve activity in responders to resynchronization therapy Europace 2008 3 374 378 10.1093/europace/eun017 18308757

[B37] Katsikis A Ekonomopoulos G Papaioannou S Kouzoumi A Koutelou M Reversible reduction of cardiac sympathetic innervation after coronary artery bypass graft surgery: an observational study using serial iodine 123-labeled meta-iodobenzyl-guanidine (MIBG) imaging J Thorac Cardiovasc Surg 2012 3 210 216 10.1016/j.jtcvs.2012.03.005 22487439

[B38] Nishioka SA Martinelli Filho M Brandao SC Giorgi MC Vieira ML Costa R Mathias W Meneghetti JC Cardiac sympathetic activity pre and post resynchronization therapy evaluated by 123I-MIBG myocardial scintigraphy J Nucl Cardiol 2007 3 852 859 10.1016/j.nuclcard.2007.08.004 18022112

[B39] Nagamatsu H Momose M Kobayashi H Kusakabe K Kasanuki H Prognostic value of 123I-metaiodobenzylguanidine in patients with various heart diseases Ann Nucl Med 2007 3 513 520 10.1007/s12149-007-0062-7 18030583

[B40] Hussein AA Saliba WI Martin DO Bhargava M Sherman M Magnelli-Reyes C Chamsi-Pasha M John S Williams-Adrews M Baranowski B Dresing T Callahan T Kanj M Tchou P Lindsay BD Natale A Wazni O Natural history and long-term outcomes of ablated atrial fibrillation Circ Arrhythm Electrophysiol 2011 3 271 278 10.1161/CIRCEP.111.962100 21493959

[B41] Saliba W Wazni OM Sinus rhythm restoration and treatment success: insight from recent clinical trials Clin Cardiol 2011 3 12 22 10.1002/clc.20826 21259273 PMC6652478

[B42] Wazni O Wilkoff B Saliba W Catheter ablation for atrial fibrillation N Engl J Med 2011 3 2296 2304 10.1056/NEJMct1109977 22168644

